# Psychoeducational group intervention for intellectually able adults with autism and their close relations (Prisma) – an open feasibility study

**DOI:** 10.1186/s12888-022-04134-4

**Published:** 2022-08-19

**Authors:** Nathaniel Hidalgo, Douglas Sjöwall, Hanna Agius, Caroline Byström, Annika Brar, Jacqueline Borg, Tatja Hirvikoski

**Affiliations:** 1 Centre for Psychiatry Research, Department of Clinical Neuroscience, Karolinska Institutet, Stockholm Health Care Services, R5:02 Stockholm, Sweden; 2 Region Stockholm, Stockholm, Sweden; 3 Department of Women’s and Children’s Health, Center for Neurodevelopmental Disorders at Karolinska Institutet (KIND), Pediatric Neuropsychiatry Unit, Karolinska Institutet, Stockholm, Sweden; 4 Habilitation and Health, Stockholm Health Care Services, Region Stockholm, Stockholm, Sweden; 5 Center for Psychiatry Research, Center for Neurodevelopmental Disorders at KarolinskaInstitutet (KIND), CAP Research Center, Region StockholmGävlegatan 22B, SE-11330 Stockholm, Sweden; 6 Psychiatric Services, Stockholm Health Care Services,, Region Stockholm, Stockholm, Sweden; 7 Modigo AB, Odengatan 69, 113 22 Stockholm, Sweden

**Keywords:** Autism spectrum disorder, Family members, Adults, Intervention, Treatment

## Abstract

**Background:**

Autism spectrum disorder (ASD) in adulthood is associated with severe impairments in functioning and poor health, while ASD is also affecting close relations. Accessible first-line interventions addressing the complex clinical needs and care coordination are lacking.

**Methods:**

This study investigated the feasibility and preliminary effects of a new psychoeducational intervention (Prisma) developed for intellectually able adults with ASD and their close relations in an outpatient setting. The manualized Prisma intervention consist of four weekly group sessions guided by trained group leaders and providing information about autism, support, and services. Feasibility was examined through treatment completion rate and group-level comparisons between intervention completers and non-completers (Student’s t-test, Fisher’s exact test, and Pearson’s chi-squared test). Perceived treatment credibility was investigated by within-group comparisons of participant’s self-ratings from pre-intervention to post-intervention, as well as by group leaders’ ratings using an adjusted questionnaire. Treatment satisfaction was examined quantitatively regarding the session evaluations (Student’s t-tests), as well as by a qualitative thematic analysis of participants’ feedback. Preliminary efficacy was studied using paired t-tests (pre- and post-intervention).

**Results:**

Completion rate was 77% (*n* = 71 of the 92 adults with ASD) and 73% (*n* = 69 of the 94 close relations), respectively. Participants considered Prisma to be an acceptable intervention indicated by increases in treatment credibility and expectations from pre- to post-intervention. The group leaders reported treatment credibility in the same range as the participants. Both autistic adults and their close relations reported good treatment satisfaction for each session, while the qualitative thematic analysis indicated that Prisma could be improved by enhancing active participation. This participant feedback will be used to further improve the intervention for an upcoming RCT. Preliminary analyses of effects showed promising results with an increase in knowledge of ASD and some indications for improvements in relationship quality, mental health, quality of life, acceptance of diagnosis and burden of care.

**Conclusions:**

Overall, results indicate that the Prisma is a feasible and acceptable first-line intervention in outpatient services. Randomized controlled trials are needed to further corroborate the evidence base of this novel intervention.

**Trial registration:**

Clinicaltrials.org NCT0446097, retrospectively registered July 8th 2020.

**Supplementary Information:**

The online version contains supplementary material available at 10.1186/s12888-022-04134-4.

## Background

Autism spectrum disorder (ASD) is a neurodevelopmental disorder characterized by difficulties in social communication and repetitive and restricted behaviors with a prevalence of around 1–1.5% [1, 2]. ASD is associated with severe impairments in functioning that negatively affect major life areas such as education, work, and social relations, even among intellectually able individuals [3, 4]. Although at high risk of poor mental health [5] including suicidal behaviors [6] and poor physical health [7], adults with ASD report difficulties in accessing health care for treatable and common health conditions [8, 9]. Furthermore, knowledge and awareness of ASD may still be limited also among professionals [10]. Evidence-based interventions for autistic adults are still lacking [11], and the burden of care often remains high for the family members, even after the autistic individual reaches adulthood [12]. For a feasible health care process, first-line interventions addressing the complex clinical needs and care coordination is needed for adults with ASD and their close relations.

Psychoeducation provides structured, educational information about the condition and available services [13]. Rather than a one-way communication, psychoeducation should promote active participation by including communication and reflections, and an opportunity to share experiences. Stepped-care models suggest giving patients interventions in different phases, starting first with less demanding and more universal interventions [14]. Based on this, general psychoeducation can be given as a first-line intervention to improve accessibility and active participation in the patients’ own health care processes, while more demanding individualized interventions constitute later steps. A few studies of psychoeducational interventions including adolescents with ASD have indicated good feasibility [15, 16] but there is a lack of studies for adults. Furthermore, not only adults with ASD but also their close relations struggle to get sufficient support [17]. Close relations supporting autistic adults report high levels of worry, depression, anxiety and stress, and poor quality of life [18]. Including close relations in psychoeducational interventions targeting individuals with other psychiatric and neurodevelopmental diagnoses has been shown to lead to a better understanding of each others’ situation and improve communication [19, 20]. Studies including family members of adolescents with ASD have indicated improved ASD knowledge in parents [21]. Psychoeducational interventions directed towards adults with ASD that involve their close relations are becoming more common. However, a major issue is that these interventions have not yet been systematically evaluated [22, 23] limiting our knowledge regarding their feasibility and efficacy.

The novel psychoeducative intervention Prisma was developed to provide information regarding ASD and the available health care and societal support for individuals with ASD and their close relations. Prisma was designed to be a first-line intervention in a stepped-care process in outpatient settings. The overarching objective of this study was to determine whether the Prisma program is feasible in a clinical setting and thus suitable for further efficacy evaluation (i.e. randomized controlled trial), and how the program could be improved based on the participant feedback. The primary aim of the study was to evaluate the feasibility by investigating treatment completion, and acceptability (what participants and course leaders think) of the Prisma program in a clinical outpatient context. A secondary aim was to study preliminary effectiveness.

## Methods

This was an open feasibility study in a clinical outpatient context using a mixed-methods approach (i.e., both qualitative and quantitative methods), including adults with ASD and their close relations. The study was approved by the Regional Ethics Committee in Stockholm (2017/1065–31/1). All participants with ASD as well as the participating close relations gave their written informed consent before inclusion in the study. The study adhered to the CONSORT 2010 Checklist and was registered at Clinicaltrials.org (NCT04460976).

### Intervention

Prisma was developed by a group of experienced clinicians with different health care professions from outpatient clinics (two of the authors were coordinating the group: NH, AB). Prisma was primarily designed to be the first intervention after the establishment of an ASD diagnosis at any age in adulthood, and for young adults transitioning into adult services. The aspiration was to make Prisma into an accessible intervention for intellectually able (defined as not having intellectual disability) adults with ASD in outpatient services, and their close relations. The goal is to increase knowledge about autism, as well as provide information on how to access further services. Another important aspect is to enhance active participation and increase relevance for each individual by providing opportunities to ask questions and reflect on individual needs, as well as meet peers with similar experiences.

Prisma is a group-based face-to-face intervention that can be administered by health care professionals with experience of adults with ASD by following the Prisma manual. One to two clinicians (group/course leaders) give the intervention at the clinic and each session is administered by use of a digital slide show with detailed group leader instructions. Participants receive a personal workbook including supporting instructions and spaces to make notes. The structured mapping of own needs in relation to the general session content is registered at the end of each session, using a work sheet included in the workbook. The intervention consists of four weekly 2-h sessions (including breaks, time for questions, and structured mapping of individual needs). For descriptions of content, themes, and focus of the four Prisma sessions, see Table 1.

**Table 1:** Descriptions of the themes and specific content of the four sessions included in Prisma

Session theme	Summary of the contents
1. *Introduction to ASD*	- Basic information about ASD. Prevalence, diagnostic criteria, and causes - Heterogeneity, neurodiversity and gender differences - Obstacles and strengths associated with ASD
2. *Different ways of functioning*	- Social interaction and communication: Social reciprocity, non-verbal communication, developing and maintaining relationships - Behaviors and interests: Repetitive behaviors, need for routines, intensive interests, and sensitivity to sensory input - Obstacles and strengths associated with ASD
3. *Well-being in everyday life*	- Basic needs: nutrition, sleep, and exercise - Stress: What is stress and why do people with ASD often experience more stress? Different ways of preventing stress - Occupation: Plan and prioritize tasks, social interaction, unwritten rules, sensory overload, etc. Obstacles and strengths associated with ASD - At home: Cleaning, cooking, etc. What can be difficult? - What kind of changes can I make myself and in which areas might I need help?
4. *Who can provide support with ASD-related challenges?*	- Support from society to adults with ASD: housing support, laws regulating services and support, financial support, etc - Support for work, employment, and studies - Driving license and ASD - Psychological and physical health - Habilitation services - Non-governmental interest organizations, getting in touch with similar others online or in real life, and links for more information

### Treatment fidelity

To increase treatment fidelity, a half-day training course, including an introduction to the intervention and course contents were given to course leaders. Also, course leaders received on-going support from project coordinators (via email, digital platform, telephone, or visits at the respective clinic/center) throughout the study, regarding all issues related to the intervention as well as the course leader’s role. All study and intervention materials were thoroughly structured and made available to the course leaders via a digital platform. Furthermore, the course leaders had access to a manual containing all the parts that were included in the course leader training, answers to FAQs, a structured checklist for time planning, and ready-made suggestions for administration.

### Participants and recruitment

Information about how to participate in Prisma was given to patients at each clinic through information brochures in the waiting rooms and/or by clinicians. All patients that expressed interest in participation were contacted by one of the course leaders. A structured screening interview including information about the content of Prisma, as well as a brief assessment of inclusion and exclusion criteria, was conducted by telephone or at the clinic. Individuals who fulfilled the requirements were invited to an information meeting with their close relations where they received more information about the intervention, gave consent for participation, and completed the baseline measurement. An additional individual and final ascertainment of eligibility was performed with each patient and his or her close relations to ensure that the prerequisites for participation were met. The eligibility was based on follow-up on screening interview and relevant questionnaires (The Hospital Anxiety and Depression Scale [HADS]), the patient's own description of his or her current situation and medical records.

The data collection was conducted in 2017 at eight adult outpatient psychiatric clinics and four habilitation clinics^1^ in the Stockholm area. In total, 13 groups received the psychoeducational intervention Prisma. Each group included approximately 10–15 participants (M = 13 for this study) with ASD and 1–2 close relations per participant.

### Inclusion and exclusion criteria

For inclusion, patients had to meet DSM-IV and/or DSM 5 criteria for ASD and/or ICD-10 criteria for one of the ASD diagnoses under F84, assessed within the Swedish health care system before the study participation. Both patients and the close relations had to be 18 years or older. Sufficient knowledge of the Swedish language was required to understand the course contents. Close relations could be a parent, sibling, partner, friend, or whomever the participant with ASD thought of as a close relation. Having ASD or other diagnoses were not considered exclusion criteria for close relations.

Exclusion criteria for the participants with ASD were not being able to participate with a close relation, intellectual disability, mental or psychosocial instability to a degree that made participation impossible as judged by the course leader or experienced health care professionals (i.e. severe psychiatric comorbidity such as ongoing substance use disorder, manic episodes, psychosis, and acute suicidality). Further reasons for exclusion were the inability to participate in a group, or severe life situations (e.g. homelessness). Parallel treatments and interventions like pharmacological or occupational therapy were not an exclusion criterion. No changes in inclusion and exclusion criteria were made during the study.

### Measures

#### Demographic data

Case histories and sociodemographic data for participants with ASD were extracted from medical records. The participants also completed a questionnaire “Current Life Situation Form” covering demographic information and current stressors in different areas of life [24]. A modified version of this questionnaire was used to assess demographic characteristics of the close relation. ASD symptoms were measured using the Ritvo Autism Asperger Diagnostic Scale-14 screen (RAADS-14) [25]. Background and demographic data are described in Table 2 for adults with ASD and in Table 3 for close relations.

**Table 2:** Baseline characteristics for autistic Prisma participants and comparison of intervention completers to non-completers

	**All *n* = 92**	**Completers *n* = 71**	**Non-Completers *n* = 21**	**χ2/*t* value**
**Mean age (range) (SD)**	31.4 (18–64) (11.8)	32.7 (18–64) (12.0)	27.1 (18–50) (9.9)	1.98 ns
**Gender female *n* (%)**	45 (48.9%)	31 (43.6%)	14 (66.7%)	3.43 ns
**Highest education *n* (%)**				
9 year compulsory school or less	17 (19.1%)	12 (17.1%)	5 (26.3%)	2.04 ns
High school	53 (59.6%)	41 (58.6%)	12 (63.2%)	
University degree (or higher)	19 (21.3%)	17 (24.3%)	2 (10.5%)	
**Occupation *n* (%)**				
Employed/student	33 (37.1%)	24 (34.3%)	9 (47.4%)	1.10 ns
Unemployed	56 (62.9%)	38 (65.7%)	10 (52.6%)	
**Partner *n* (%)**	37 (43.5%)	32 (47.1%)	5 (29.0%)	1.72 ns
**Years diagnosed with ASD *n* (%)**				
≤1 years:	60 (67.4%)	49 (70.0%)	11 (57.9%)	1.00 ns
> 1 years:	29 (32.6%)	21 (30.0%)	8 (42.1%)	
**Comorbidity *n* (%)**				
*Other neurodevelopmental disorders*	44 (50.0%)	34 (48.6%)	10 (55.6%)	0.28 ns
*Other psychiatric disorders*	54 (61.3%)	45 (64.3%)	9 (50%)	1.23 ns
*No neurodevelopmental or psychiatric disorders*	15 (17.0%)	12 (17.1%)	3 (16.7%)	0.01 ns
*Self-reported physical illness/diseases*	35 (39.8%)	26 (37.7%)	9 (47.4%)	0.58 ns
**Other treatments**	37 (41.6%)	32 (45.7%)	5 (26.3%)	2.32 ns
**RAADS-14 screen mean (SD)**	27.4 (9.4)	26.9 (9.74)	29.2 (8.2)	0.94 ns

Note: *ns* non-significant, *SD* Standard deviation, *RAADS* The Ritvo Autism Asperger Diagnostic Scale

The percentages are counted by entering the number of individuals having data for that variable in the denominator

**Table 3:** Baseline characteristics for close relations who started Prisma, and comparison of intervention completers to non-completers

	**All *n* = 94**	**Completers *n* = 69**	**Non-Completers *n* = 25**	**χ2/*t* value**
**Mean Age (range) (SD)**	52.1 (17–86) (13.5)	53.2 (18–86) (12.8)	49.0 (17–75) (15.4)	1.28 ns
**Gender female *n* (%)**	58 (61.7%)	44 (63.8%)	14 (56.0%)	0.47 ns
**Relation to the participant with ASD* n* (%)**	Parent: 62 (67.4%)	Parent: 48 (69.6%)	Parent: 14 (60.9%)	5.07 ns
	Partner: 21 (22.8%)	Partner: 17 (24.6%)	Partner: 4 (17.4%)	
	Other: 9 (9.8%)	Other: 4 (5.8%)	Other: 5 (21.7%)	
**Highest education *n* (%)**				
9 year compulsory school (or less)	8 (8.7%)	7 (10.1%)	1 (4.3%)	2.71 ns
High school	39 (42.3%)	26 (37.7%)	13 (56.5%)	
University degree (or higher)	45 (48.9%)	36 (52.2%)	9 (39.1%)	
**Occupation *n* (%)**				
Employed/student	67 (72.8%)	51 (73.9%)	16 (69.6%)	0.17 ns
Unemployed	11 (12.0%)	8 (11.6%)	3 (13.0%)	
Retired	14 (15.2%)	10 (14.5%)	4 (17.4%)	
**Disability n (%)**				
*ASD*	3 (3.3%)	3 (4.4%)	0	1.05 ns
*Other neurodevelopmental disorders*	8 (8.8%)	5 (7.4%)	3 (13.0%)	0.69 ns
*Other psychiatric disorders*	8 (8.8%)	6 (8.8%)	2 (8.7%)	0.00 ns
*No neurodevelopmental or psychiatric disorders*	75 (82.4%)	57 (83.8%)	18 (78.3%)	0.37 ns
*Physical illness/diseases*	3 (3.3%)	1 (1.5%)	2 (8.7%)	2.76 ns

Note: *ns* non-significant, *SD* Standard deviation

#### Primary outcome: feasibility

Treatment completion was a central outcome for evaluating if Prisma would be a feasible intervention in a clinical setting for adults with ASD and their significant others. Moreover, since adults with ASD were not part of the group that developed Prisma, an important goal for this study was to gather feedback and experiences (treatment acceptability) from participants with ASD and their close relations to further develop Prisma. Outcome measures were gathered through self-ratings before, during, and after Prisma. In addition, for preliminary estimation of treatment effects, self-rating questionnaires were administered at baseline, i.e. 1–2 weeks before the intervention started, and post-intervention, i.e. 1–2 weeks after the last session.

*Treatment completion* was defined as the proportion of participants who completed the intervention. To be regarded as a completer, the participant had to attend at least 3 out of 4 course sessions.

*Acceptability* was addressed by measuring credibility, satisfaction, and safety. Treatment credibility was measured with an adjusted version of the Treatment Credibility Scale (TCS) [26]. TCS was administered at baseline (Cronbach’s alfa 0.81, *n* = 138) and after the last session to participants and course leaders. The TCS includes five items in a 10-point visual analogous scale (VAS). High values indicate high credibility. Treatment satisfaction was evaluated after each session and after the intervention was completed. The Session Evaluation Form (SEF) is a modified version of the Evaluation Questionnaire [19, 27]. SEF consists of five statements rated 0–4 on a Likert scale. Three of the statements target the respondent’s appraisal of the content of the specific lecture. The other two assess the participant’s experience of taking part in group discussions/exchange of experiences and could also be answered “not applicable” if the participant did not share or discuss experiences. At the end of each form, the participants could write comments about the session.

A modified 12-item version of the Patient Evaluation Form (PEF) [28] was distributed at the end of the last session regarding the participant appraisal of the course as a whole. Six items are scored on a Likert scale ranging from 0–4 (Cronbach’s alfa 0.78, *n* = 132). Four items are open-ended questions for participants to further develop their answers (“How did the intervention help me?”; “How can the intervention improve?”; “What could I have done differently?”; “Is there anything else you want to share about the intervention?”). For the open-ended questions in the PEF, a qualitative thematic analysis was performed [29]. As the non-completers only participated to a limited extent and did not assess the full content of the intervention, only data from completers were included in the main thematic analysis. However, a separate analysis was conducted on non-completers. As participants who answered the open-ended questions at times gave more than one answer, the percentage in Table 6 reflects the proportion of answers rather than individuals. To be considered a theme, a minimum of four similar answers had to be present. One of the authors (DS) analyzed and categorized the answers in themes and another author (NH) confirmed them. The agreement was very high and the few deviating analyses were discussed and placed in the theme’s authors agreed upon. Yet another author (TH) reviewed and confirmed the categorizations. After a consensus discussion with all three researchers (NH, DS, TH), slight changes were made to the description of the themes to clarify what they reflected.

Adverse events were defined as spontaneous oral complaints or instances when patients stated that they experienced negative or unwanted effects during the intervention period. Serious adverse events were defined as events that involved hospital care/hospitalization, due to the intervention. Adverse events and serious adverse events were recorded in the case report form (CRF) folder, and it was judged by the research group if these were associated with the intervention.

#### Preliminary effectiveness outcomes

All scales that measure preliminary effectiveness were administrated to participants before and after the intervention except for the Burden Assessment Scale (BAS) [30] which was only filled in by close relations.

*Acquired knowledge of ASD and support and services* was measured using the ASD 20 Questions (Additional file 1). ASD 20 Questions is a knowledge quiz with 20 true/false/don’t know scored items created for this study. High values indicate more acquired knowledge. Moreover, a separate question was included where participants were asked to list all support and treatment interventions helpful for adults with ASD and their close relations. Similar knowledge quizzes have been used in previous studies [15, 27]. Internal consistency of the quiz using Kruder-Richardson 20, at pre-intervention was 0.78 (*n* = 138).

*Relationship quality* was measured both from the perspective of the participant with ASD and the close relation with The Questions About Family Members (QAFM) [31]. The QAFM comprises four subscales: (1) Critical Remarks Cronbach’s alfa pre-intervention 0.86 (*n* = 131) (2) Emotional Over-involvement Cronbach’s alfa pre-intervention 0.74 (*n* = 132) (3) Perceived Criticism Cronbach’s alfa pre-intervention 0.51 (*n* = 132) and (4) Perceived Emotional Involvement Cronbach’s alfa pre-intervention 0.43 (*n* = 135). The scale contains 30 items, which are scored on a 5-point Likert scale. Low scores on the first three subscales, and high scores on the fourth subscale, are indicative of a good quality of relationship.

*Mental health.* The Hospital Anxiety and Depression Scale (HADS) [32] was used to measure mental health on the two subscales: “depression” Cronbach’s alfa pre-intervention 0.84 (*n* = 139) and “anxiety” Cronbach’s alfa pre-intervention 0.88 (*n* = 138). The subscales contain seven items each and were scored on a 0–3 Likert scale with a maximum score of 21 points. Higher scores indicate high symptoms of anxiety and/or depression. Furthermore, two items from the PEF covering participants’ well-being before and after the intervention on a Likert scale of 1–10 (“How would you rate your well-being before the intervention?”, “How would you rate your current well-being”) was used to measure general well-being. High values indicate high well-being.

*Quality of life* was measured using the Satisfaction With Life Scale (SWLS) [33]. SWLS contains five items, scored on a 7-point Likert scale Cronbach’s alfa pre-intervention 0.91 (*n* = 139). High values indicate higher satisfaction with life.

*Acceptance of diagnosis* was measured using adapted version of the Acceptance and Action Questionnaire – II [34], “What I think about my diagnosis” for adults with ASD and “What I think about my close relation’s diagnosis” for close relations. Cronbach’s alfa at pre-intervention was 0.86 for participants with ASD (*n* = 68) and 0.87 (*n* = 68) close relations about the adult with ASD’s diagnosis. Both questionnaires contain 7 items, scored on a 7-point Likert scale where lower values indicate higher acceptance.

*The burden of care* on close relations was assessed using the Burden Assessment Scale (BAS) [30] consisting of 19 items scored on a 4-point Likert scale Cronbach’s alfa at pre-intervention 0.87 (*n* = 68). High values indicate a greater burden. BAS was used to measure to what extent the close relations experienced a subjective (e.g. emotional distress) and objective (e.g. economic consequences) burden of care.

### Statistical analysis

Statistical outliers in all outcome measures were screened using boxplots in SPSS version 27. The few extreme outliers (1st/3rd Quartile ± 1,5) that were identified did not significantly affect the results and were therefore retained in the subsequent analyses. Outcome data were approximately normally distributed. The main statistical analyses regarding feasibility and efficacy-related measures were performed on all participants attending at least 3 out of 4 sessions and who had completed pre- (T1) and post-measurement (T2).

Comparisons between completers and non-completers were performed on baseline data using unpaired t-tests, Fisher’s exact test, and Pearson’s chi-squared test to detect possible predictors of drop-outs. Unpaired t-tests were used to examine differences post Prisma between individuals with ASD and close relations on the SEF and the PEF. Paired t-tests (pre and post) were used to test preliminary efficacy both for individuals with ASD and the close relations for all outcomes. The effect size was interpreted according to Cohens *d*: 0.2 = small effect size, 0.5 = medium effect size and 0.8 = large effect size [35].

## Results

### Feasibility

#### Treatment completion

**Fig. 1:**
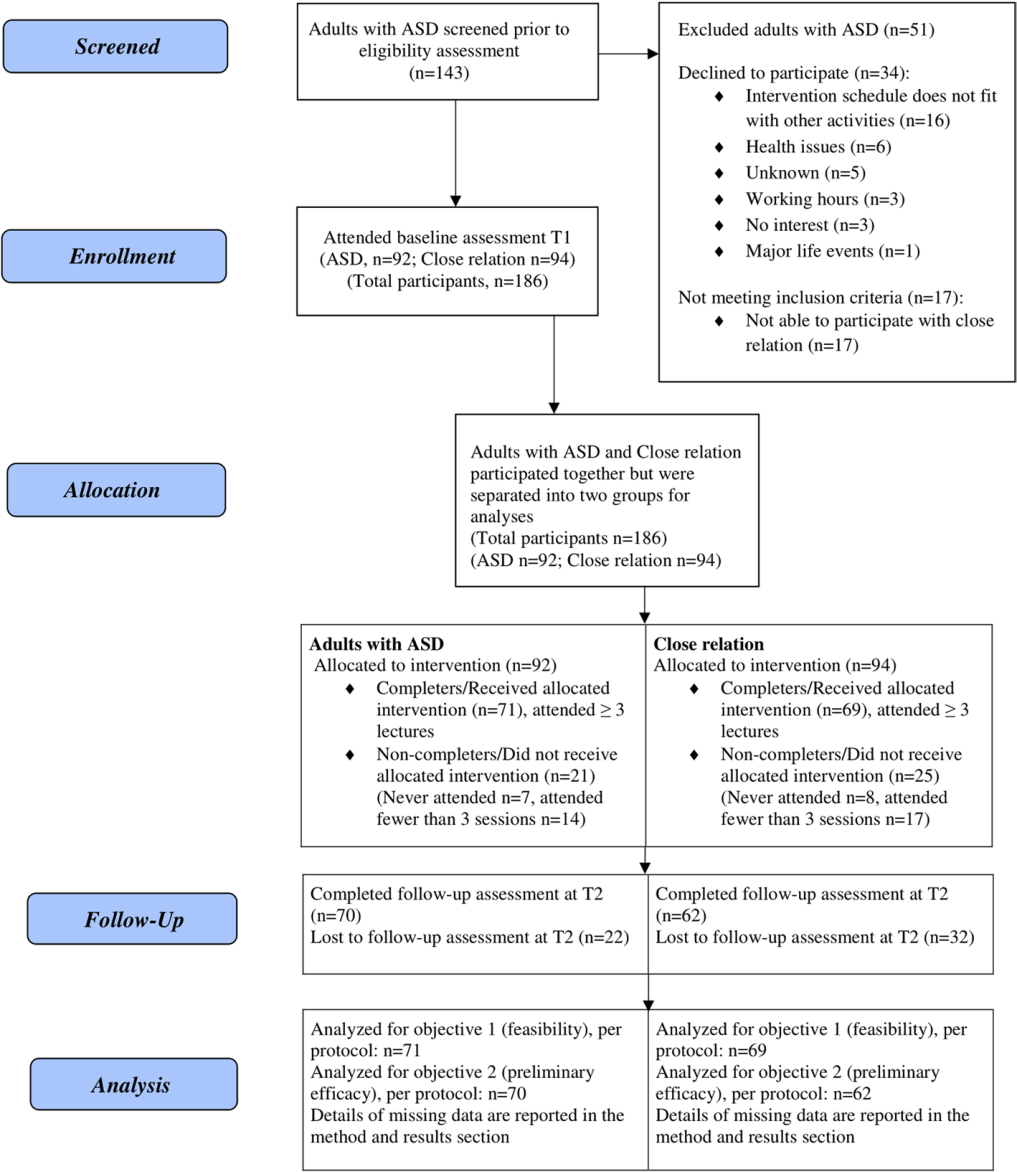
Flowchart for all participants and reasons for dropping out during the study

The flow of study participants is presented in Fig. 1. A total of 143 adults with ASD were screened for eligibility to participate. Out of these, 12% (*n* = 17) could not be included in the study due to that attending with a close relation was a requirement and 24% (*n* = 34) declined participation, with the most common reason for this being that the date/time of the upcoming intervention did not fit with other activities in their lives. Of the adults with ASD screened for intervention, 64% (*n* = 92) and 94 close relations were included in the intervention.

Of those enrolled in Prisma, 77% (*n* = 71out of 92) of the adults with ASD and 73% (*n* = 69 out of 94) of the close relations met the predefined criteria for completion (attending at least 3 out of 4 sessions). There were no significant differences between completers and non-completers in the background and demographic data (Table 2 for adults with ASD and Table 3 for close relations).

#### Acceptability

##### *Treatment credibility*

Both ASD participants and their close relation reported significantly higher credibility post-intervention compared to before starting (Table 4).

**Table 4:** Pre- and post-measures of treatment credibility and expectations using the Treatment Credibility Scale

Pre-intervention Mean (SD)		Post-intervention Mean (SD)	*t value*	Effect size *d (95% CI)*
Participants with ASD	7.15 (1.55) *n* = 65	7.63 (1.76) *n* = 65	2.64**	0.29 (-0.06 – 0.63)
Close relations	7.56 (1.23) *n* = 59	8.29 (1.20) *n* = 59	4.74***	0.60 (0.23 – 0.97)
Course leaders	7.03 (1.29) *n* = 13	7.15 (1.37) *n* = 13	0.49	0.09 (-0.63 – 0.86)

Note: ** *p* < 0.01, *** *p* < 0.001. Effect sizes refer to Cohen’s *d,* parentheses include the 95% confidence interval (CI)

##### *Treatment satisfaction*

All items and all sessions were rated as being satisfactory “to some extent” on average (mean 2.52—3.53 on a scale from 0–4). Levels of treatment satisfaction were the same between ASD participates and their close relations with three exceptions; these were rated higher by the close relations (effect sizes in the medium range) (see Table 5).

**Table 5:** Comparison of autistic participants’ and close relations’ session evaluations (items in the Session Evaluation Form)

	**Session 1**			**Session 2**			**Session 3**			**Session 4**		
	**ASD Mean (SD)** ***n* = 71**	**Close relations Mean (SD)** ***n* = 64**	***t-*value (df) Effect size *d***	**ASD Mean (SD)** ***n* = 66**	**Close relations Mean (SD)** ***n* = 63**	***t*-value (df) Effect size *d***	**ASD Mean (SD)** ***n* = 63**	**Close relations Mean (SD)** ***n* = 57**	***t-*value (df)**	**ASD Mean (SD)** ***n* = 67**	**Close relations Mean (SD)** ***n* = 63**	***t-*value (df) Effect size *d***
**Item 1. Increased knowledge**	3.00 (0.89)	3.18 (0.76)	1.28 (135)	2.95 (0.85)	3.13 (0.83)	1.16 (128)	3.00 (0.80)	3.10 (0.86)	0.67 (121)	2.52 (1.28)	2.86 (0.98)	1.66 (128)
**Item 2. Useful content**	2.93 (0.86)	3.38 (0.70)	3.37** (134) *d* = 0.57	3.06 (0.96)	3.47 (0.73)	2.72** (128) *d* = 0.48	3.21 (0.79)	3.45 (0.59)	1.93 (121)	3.16 (0.88)	3.43 (0.64)	1.95 (128)
**Item 3. Relevant content**	3.21 (0.88)	3.47 (0.73)	1.87 (135)	3.33 (0.88)	3.50 (0.56)	1.28 (128)	3.44 (0.69)	3.53 (0.68)	0.65 (120)	3.01 (1.07)	3.32 (0.86)	1.78 (128)
**Item 4. Helpful to share**	2.63 (1.09) N/A = 30	2.69 (1.17) N/A = 29	0.23 (75)	2.81 (1.10) N/A = 19	3.06 (1.01) N/A = 29	1.05 (79)	2.81 (1.03) N/A = 11	3.15 (0.83) N/A = 24	1.61 (83)	2.60 (1.20) N/A = 24	2.94 (1.09) N/A = 30	1.26 (74)
**Item 5. Others’ experiences helpful**	2.87 (1.02) N/A = 16	2.92 (1.01) N/A = 14	0.26 (101)	3.09 (1.01) N/A = 11	3.11 (1.09) N/A = 10	0.09 (106)	3.12 (0.87) N/A = 3	3.33 (0.83) N/A = 2	1.36 (114)	2.67 (1.05) N/A = 13	3.12 (0.96) N/A = 11	2.30* (102) *d* = 0.45

Note: Effect sizes shown for significant differences only

*N/A* Not applicable (i.e. participants did not consider this to have taken place

^*^ *p* < 0.05, ** *p* < 0.01

For all sessions, 17%—42% of the ASD participants and 42%—48% of close relations answered “Not applicable” to the statement “It was helpful to share experiences with other participants”, thus indicating that they did not participate in experience sharing during the sessions.

**Fig. 2:**
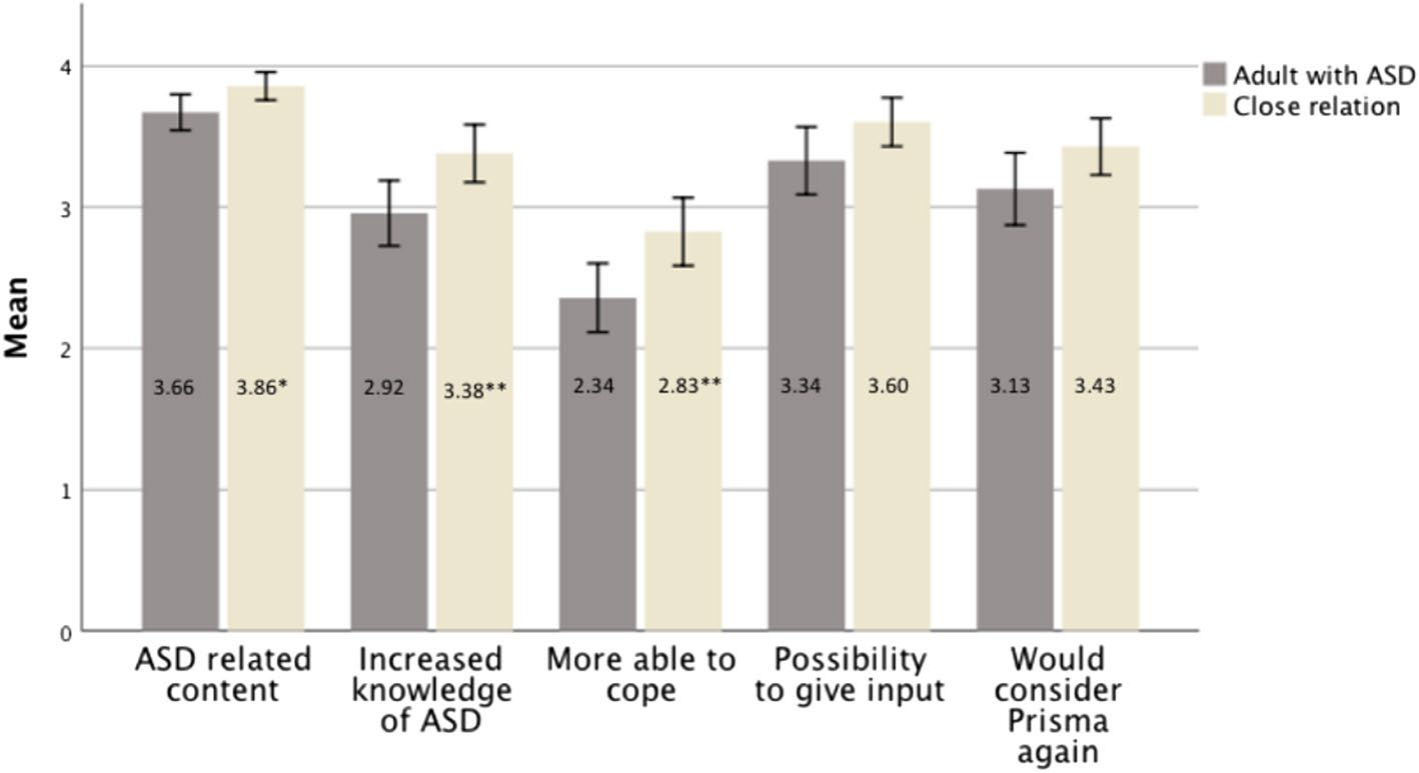
Mean values, separated for adults with ASD and close relations for different aspects of satisfaction. Note: 0=not at all, 4=yes, absolutely; *p<.05, **p<.01; Error bars represent 95% CI; Treatment satisfaction was measured using the Patient Evaluation Form after completing Prisma. Significance testing was done comparing adults with ASD to close relations

Assessment of satisfaction (PEF) of the whole course was also filled out after the last session. Overall, there was a significant difference between adults with ASD (M = 3.07, SD = 0.69) and their close relations (M = 3.42, SD = 0.44); (*t*(132) = 3.44, *p* < 0.001, *d* = 0.60). Mean scores on the individual items are presented in Fig. 2. Participants also rated the intervention as a whole (0 = “Not approved”, 1 = “Approved”, 2 = “Well approved” or 3 = “Very well approved”) with a mean score of 1.97 for participates with ASD and 2.13 for close relations.

A thematic content analysis of the participants’ answers for the open questions in the PEF is presented in Table 6.

**Table 6:** Thematic analysis of the responses to the open-ended questions regarding the whole intervention

Question	Participant with ASD	Close relation
How has the intervention been helpful?	*n* = 48 individuals Total number of answers *n* = 61 • Knowledge about ASD and where to turn for support: 31 (51%) • Acceptance of diagnosis, knowing yourself, courage, recognition: 25 (41%) • Suggestion for improvements (no new knowledge, too little interacting with others with ASD): 5 (8%)	*n* = 36 individuals Total number of answers *n* = 47 • Knowledge about ASD and where to turn for support: 22 (47%) • Understanding, solutions to problems, reduced stress: 23 (49%) • Other*:* 2 (4%)
How could the course be improved?	*n* = 48 individuals Total number of answers *n* = 61 • Interaction (discussions/questions, more presence, and examples from individuals with ASD): 23 (38%) • More information (concrete tips/individualized tips): 20 (33%) • Pedagogy (better presentation by the course leader, shorter sessions/easier content): 7 (11%) • Don’t know/satisfied: 8 (13%) • Other: 3 (5%)	*n* = 38 individuals Total number of answers *n* = 47 • Interaction(discussions/questions, more presence, and examples from individuals with ASD): 20 (43%) • More information (concrete tips/individualized tips): 10 (21%) • Pedagogy (better presentations by the course leader, shorter sessions/better content): 8 (17%) • Don’t know/satisfied: 4 (9%) • Other: 5 (11%)
Could you have done anything differently?	*n* = 35 individuals Total number of answers *n* = 35 • Asked questions: 6 (17%) • Shared own experiences: 7 (20%) • Engaged more in the material (prepare, take notes, repeat): 5 (14%) • Don’t know/satisfied: 12 (35%) • Other: 5 (14%)	*n* = 23 individuals Total number of answers *n* = 28 • Asked questions: 6 (21%) • Shared own experiences: 7 (25%) • Engaged more in the material (prepare, take notes, repeat): 5 (18%) • Don’t know/satisfied: 7 (25%) • Other: 4 (11%)
Is there anything else you would like to comment on?	*n* = 34 individuals Total number of answers *n* = 36 • Appreciation: 18 (50%) • Suggestion for improvement: 11 (31%) • Other: 7 (19%)	*n* = 28 individuals Total number of answers *n* = 29 • Appreciation: 7 (24%) • Suggestion for improvement: 14 (48%) • Other: 8 (28%)

Note: Number and % reflect the answers rather than individuals responding for each question

The major themes that emerged in these analyses suggest that the intervention both gave more knowledge as well as acceptance towards the diagnosis but that participants wanted more interactive activities. A separate analysis was performed with participants that dropped out during the intervention. The themes were similar to what was observed for completers, but as very few answers were given, these results should be interpreted with caution.

##### *Adverse events and serious adverse events*

No serious adverse events were reported. There was one adverse event that was judged to be related to taking part in Prisma. One participant with ASD reported a deteriorated mood from participating in the intervention and therefore chose not to continue. In addition, 8% (*n* = 7) of the patients with ASD reported increased levels of depression and/or anxiety, but it was not specifically reported that it was linked to taking part in the intervention. In two of these 7 cases, external causes of the deteriorating mood were specified by the participants.

##### Group leaders’ rating of treatment credibility

To evaluate the clinicians’ perspectives on the Prisma program, group leaders who administrated the intervention completed an adjusted version of the Treatment Credibility Scale (TCS, see above). Course leaders’ evaluation of Prisma was just above 7 on a 1–10 scale of the TCS both pre-and post-intervention.

### Preliminary effectiveness

Descriptive statistics and results from pre- to post-intervention for all preliminary effectiveness measures are reported in Table 7 for both participants with ASD and their close relations.

**Table 7:** Preliminary effects for all participants and separately for participants with ASD and close relations, respectively

Outcome measures	Baseline Mean (SD)	Post-intervention Mean (SD)	*df*	*t* value	Effect size *d (95% CI)*
***ASD knowledge***					
All participants	12.90 (3.82)	16.26 (3.01)	124	10.94***	*d* = 0.97 (0.71–1.12)
Participants with ASD	12.77 (3.63)	16.20 (3.54)	65	8.82***	*d* = 0.96 (0.59–1.31)
Close relations	13.03 (4.06)	16.32 (2.29)	58	6.73***	*d* = 1.00 (0.61–1.37)
***ASD knowledge—support/interventions***					
All participants	3.02 (2.68)	4.64 (3.06)	120	6.61***	*d* = 0.56 (0.30–0.82)
Participants with ASD	2.53 (2.34)	3.94 (3.09)	63	4.17***	*d* = 0.51 (0.16–0.86)
Close relations	3.56 (2.93)	5.42 (2.85)	56	5.21***	*d* = 0.64 (0.26–1.02)
***QAFM perceived criticism***					
All participants	12.79 (4.74)	12.76 (4.79)	123	0.10	*d* = 0.01 (-0.24–0.26)
Participants with ASD	12.22 (4.79)	12.25 (4.75)	64	0.07	*d* = 0.01 (-0.35–0.34)
Close relations	13.42 (4.64)	13.32 (4.81)	58	0.24	*d* = 0.02 (-0.34–0.38)
***QAFM perceived emotional involvement***					
All participants	13.41 (3.37)	13.95 (2.71)	123	2.00*	*d* = 0.18 (-0.07–0.43)
Participants with ASD	13.42 (3.95)	13.86 (2.79)	64	1.00	*d* = 0.13 (-0.22–0.47)
Close relations	13.41 (2.64)	14.05 (2.64)	58	2.26*	*d* = 0.24 (-0.13–0.60)
***QAFM perceived critical remarks***					
All participants	20.51 (7.49)	19.91 (7.25)	123	1.25	*d* = 0.08 (-0.17–0.33)
Participants with ASD	18.91 (7.34)	19.17 (7.25)	64	0.33	*d* = 0.04 (-0.31–0.38)
Close relations	22.27 (7.33)	20.73 (7.23)	58	3.12**	*d* = 0.21 (-0.15–0.57)
***QAFM emotional overinvolvement***					
All participants	19.79 (6.37)	19.40 (5.60)	123	1.01	*d* = 0.06 (-0.18–0.31)
Participants with ASD	17.25 (5.84)	17.57 (5.12)	64	0.55	*d* = 0.06 (-0.29–0.40)
Close relations	22.59 (5.75)	21.41 (5.45)	58	2.40*	*d* = 0.21 (-0.15–0.57)
***Anxiety (HADS)***					
All participants	8.78 (4.70)	8.27 (4.80)	125	2.38*	*d* = 0.11 (-0.14–0.35)
Participants with ASD	11.27 (3.88)	10.38 (4.49)	65	2.88**	*d* = 0.21 (-0.13–0.55)
Close relations	6.03 (3.94)	5.95 (4.03)	59	30	*d* = 0.02 (-0.34–0.38)
***Depression (HADS)***					
All participants	5.53 (4.06)	4.87 (3.84)	125	3.06**	*d* = 0.17 (-0.08–0.41)
Participants with ASD	7.23 (4.11)	6.52 (3.91)	65	2.04*	*d* = 0.18 (-0.17–0.52)
Close relations	3.67 (3.10)	3.05 (2.84)	59	2.45*	*d* = 0.21 (-0.15–0.57)
***Global life satisfaction (SWLS)***					
All participants	4.10 (1.51)	4.23 (1.51)	125	2.05*	*d* = 0.09 (-0.16–0.33)
Participants with ASD	3.33 (1.40)	3.53 (1.50)	65	2.12*	*d* = 0.14 (-0.20–0.48)
Close relations	4.96 (1.11)	5.00 (1.08)	58	0.59	*d* = 0.04 (-0.32–0.40)
***Acceptance of diagnosis***					
Participants with ASD	3.70 (1.31)	3.51 (1.31)	64	2.15*	*d* = 0.15 (-0.20–0.49)
Close relations	2.15 (1.03)	2.09 (0.89)	58	0.61	*d* = 0.06 (-0.30–0.42)
***Burden Assessment Scale (BAS) for close relation***					
Objective burden	0.69 (0.57)	0.71 (0.67)	58	0.28	*d* = 0.03 (-0.33–0.39)
Subjective burden	0.81 (0.57)	0.70 (0.53)	58	2.23*	*d* = 0.20 (-0.16–0.56)

Note: * p < 0.05, ** *p* < 0.01, *** *p* < 0.001

Effect sizes refer to Cohen’s *d,* parentheses include the 95% confidence interval (CI)

#### Acquired knowledge of ASD and support and services

Knowledge of ASD increased for both participants with ASD and close relations with large effect sizes. Knowledge about support and services also improved with medium-sized effects.

#### Relationship quality

Measurement on QAFM indicates small significant positive effects between pre-and post-assessments for close relations on perceived emotional involvement, critical remarks, and perceived emotional over-involvement. No significant changes were observed in participants with ASD.

#### Mental health

Both depression and anxiety symptoms significantly decreased for participants with ASD (small effect sizes) whereas there was only a significant decrease in depression for close relations (small effect size). Also, participants were asked after the intervention to rate their well-being both before and after the intervention using the PEF (see above). Both participants with ASD (pre- intervention M = 5.41, SD = 1.93, post intervention M = 5.97, SD = 1.94; (*t*(69) = 3.97, *p* < 0.001, *d* = 0.29) and close relations (pre- intervention M = 6.97, SD = 1.72, post intervention M = 7.51, SD = 1.66; (*t*(62) = 3.65, *p* < 0.001, *d* = 0.32) reported improved well-being post-intervention.

#### Quality of life

There was a small significant effect indicating that participants experienced a better quality of life after receiving the intervention. However, this effect was only significant in patients with ASD and not their close relations when analyzed separately for the two groups.

#### Acceptance of diagnosis

Participants with ASD reported slightly better acceptance of their diagnosis after receiving the intervention.

#### *The burden of care* on close relations

A small decrease in the subjective burden of care was observed for close relations while the objective burden was unchanged from pre- to post-intervention.

## Discussion

This study evaluated the feasibility of the novel manualized psychoeducational intervention Prisma for adults with ASD and their close relations, thus addressing the need for the development of scientifically evaluated interventions [11]. The vast majority completed Prisma and overall, it was perceived as an acceptable intervention by both adults with ASD and their close relations. Moreover, preliminary analyses of effectiveness indicated increased knowledge and well-being. However, this study also identified areas of improvement such as the intervention’s ability to enhance active participation.

### Feasibility

#### Treatment completion

Of the adults with ASD who were included 77% (*n* = 71 of 92) completed the intervention (attended ≥ 3 lectures), which was slightly more than for the close relations 73% (*n* = 69 out of 94). Compared to the general attrition in regular psychiatric services, which varies substantially from 26 to 82% [36] the attrition in Prisma was in the lower range. Regarding adults with ASD, there is a lack of studies specifically reporting attrition; however, the observed attrition in the current study was similar to what has been reported for autistic adolescents [15]. This suggests that the attrition rate for the Prisma intervention was acceptable, despite that the study was conducted in a clinical outpatient context not only recruiting from the clinics’ usual patient base, but also administered by staff members at these clinics. We speculate that one of the reasons for the high treatment completion was participating with a close relation. Participating with a close relation may provide an opportunity for supporting each other in overcoming obstacles for participation during the intervention and afterwards in the continued care process. Another important factor might have been that the clinical staff perceived Prisma as creditable. Again, this should be considered as promising for possible future implementation as these were clinicians in the healthcare settings where Prisma is intended.

The background and demographic characteristics of the included sample (e.g. educational level, or psychiatric comorbidity) corresponded well to previous studies on clinical samples [37–39] thus indicating that the results may generalize to similar clinical outpatient contexts for adults with ASD. The gender balance was 49% female, i.e. relatively close to the male-to-female ratio 3:2 reported for adult patients (18 years of age and older) in the Stockholm Region between 2012–2016, who received an ASD diagnosis without intellectual disability according to the Center for Epidemiology and Community Medicine, Region Stockholm (personal communication, 21 May 2021).

#### Acceptability

An important outcome of Prisma was acceptability (i.e., how participants react to the intervention). One challenge was building general trust for health care providers as previous research has shown that knowledge and awareness of ASD can be limited also among professionals [10] and autistic individuals find it hard to access mental health support and experience high levels of stigma [8]. The increase in perceived credibility from pre- to post-intervention should therefore be regarded as important, especially as treatment credibility is associated with post-treatment outcomes [40]. Furthermore, the reported treatment satisfaction was high in both autistic adults and their close relations. However, the close relations rated certain aspects as more satisfactory than adults with ASD. Based on the qualitative analysis of reflections from participants, the intervention could be improved especially for adults with ASD who wanted more possibilities to share their own and listen to others’ experiences. High treatment satisfaction together with an expressed will to interact with others in similar situations, suggests that a group format including individuals with ASD is a feasible and acceptable way of delivering psychoeducation. This is in line with research showing that communication is more effective and motivating when autistic individuals share information [41]. Moreover, close to half of the participants in this study reported that they did not participate in the discussions. This indicates that Prisma needs to be revised to promote active participation in the intervention and the subsequent health care process.

### Preliminary effectiveness

A secondary aim was to investigate the preliminary effects of Prisma. Knowledge of ASD and of available support/interventions improved for both groups with medium to large effect sizes, which is comparable to what has been reported for adults with ADHD and their close relations [19], as well as adolescents and young adults with ASD [15] after receiving psychoeducation. Means and individual scores of correct answers pre-and post-intervention were on the higher end of the distribution for knowledge of ASD, thus indicating possible ceiling effects.

Regarding the relationship quality, close relations perceived the relationship with the person with ASD as more positive after the intervention regarding emotional involvement, criticism, and emotional over-involvement. Also, close relations reported feeling a decrease in the subjective burden of care (i.e. emotional distress), despite the remaining objective consequences such as negative effects on the economy or the close relations’ time and activities. However, from the perspective of the individuals with ASD, we saw no changes in relationship quality, thus indicating the need for further changes to the intervention to enhance mutual active participation. However, preliminary results related to relationship quality (perceived criticism and perceived emotional involvement) should be interpreted with caution as these scales showed poor internal consistency.

Preliminary analysis of depression and anxiety symptoms showed significant decreases with small effect sizes. Even though participants generally wanted more possibility to interact with others, we speculate that it is possible the intervention in its current structure also have led to recognition between participants, a sense of belonging, acceptance, and therefore, fewer symptoms of depression and anxiety. This is also supported by that large number of the answers from adults with ASD indicated that the intervention was helpful by increasing the acceptance of diagnosis, courage, recognition, and self-awareness (see Table 6). Similarly, the decrease in depression and anxiety symptoms was consistent with the increase in reported general well-being.

### Future revision of the Prisma intervention

Since the adults with ASD and their close relations were not part of the group that developed Prisma, it was crucial to conduct the present feasibility study so that we could further improve the acceptability and accessibility of the Prisma program based on the participants’ feedback. In line with this feedback, an extensive revision was carried out after the completion of this study. The opportunities to exchange experiences were increased and updated including structured discussions in small groups with predetermined topics. These discussions now also include a method (“communication traffic light system”) for how participants can choose to participate to the extent that they prefer. Furthermore, to increase treatment acceptability, a revision was done with an increased focus on strength to balance the focus on ASD-related obstacles, following reflections from participants with ASD in the current study. The course leader manual was updated including more concrete ways in which they could support the participants. Other updates have been made emphasizing that course leaders need to conduct individual post-intervention follow-ups regarding a plan for the continued health care process after this first-line group-based psychoeducational program. How these changes are received and how this will affect the effectiveness will be evaluated in an ongoing RCT.

#### Limitations

The results of this study need to be considered in light of some limitations. First, the relatively small size of the group of non-completers may have limited our ability to detect significant differences. This will however be addressed in the next study phase including a larger sample. Second, this study did not include any controls and we cannot be sure that the observed preliminary effects are related specifically to Prisma. Third, most questionnaires used in this study are not adapted for an adult ASD population which may have a negative effect on reliability and validity of the measures. Forth, as this study was conducted in a clinical context, and due to resources allocation, we were not able to include a long-term follow-up of preliminary effectiveness.

## Conclusions

In summary, the overall patterns in the results indicate Prisma to be feasible and the preliminary effects are promising. Areas of improvement and limitations were identified, which have been addressed in the revised Prisma program used in forthcoming study phases. Hence, we feel encouraged to make the suggested adjustments and continue with a randomized controlled trial.

## Supplementary Information

**Additional file 1.** CONSORT 2010 checklist of information to include when reporting a pilot or feasibility trial*

## Data Availability

The datasets generated and/or analyzed during the current study are not publicly available due to the regulations in the ethical permission for this study. The intervention materials (in Swedish) are made available for research purposes from the corresponding author.

## Abbreviations

ADHD: Attention-Deficit / Hyperactivity Disorder
ALF: Region Stockholm funds for clinical research
ASD: Autism Spectrum Disorder
BAS: Burden Assessment Scale
CONSORT: Consolidated Standards of Reporting Trials
CRF: Case Report Form
DSM-5: Diagnostic and Statistical Manual of Mental Disorders Fifth Edition
FAQ: Frequently asked questions
HADS: The Hospital Anxiety and Depression Scale
ICD-10: International Classification of Diseases Tenth Revision
PEF: Patient Evaluation Form
QAFM: The Questions About Family Members
RAADS-14: Ritvo Autism Asperger Diagnostic Scale fourteenth edition
RCT: Randomized Controlled Trial
SEF: Session Evaluation Form
SPSS: Statistical Package for the Social Sciences
SWLS: Satisfaction With Life Scale
T1: Pre-intervention measurement
T2: Post-intervention measurement
TCS: Treatment Credibility Scale
VAS: Visual Analogous Scale

## Footnotes

1 Habilitation clinics are a specialized form of healthcare directed to patients with ASD and other enduring disabilities where the goal is to attain, keep, or improve functioning and participation in their community. The Stockholm's healthcare services (like several other regions in Sweden) is organized so that patients with enduring disabilities receive support from habilitation services for functional impairments, and from psychiatric services for comorbid mental illness.
