# Supplementary material for: Psychoeducational group intervention for intellectually able adults with autism and their close relations (Prisma) – an open feasibility study
Source: BMC Psychiatry. 2022 Aug 19;22:556. doi: 10.1186/s12888-022-04134-4 (PMC9389708; doi:10.1186/s12888-022-04134-4)
Supplement: Supplementary file 1 — Additional file 1. CONSORT 2010 checklist of information to include when reporting a pilot or feasibility trial* [file 12888_2022_4134_MOESM1_ESM.doc]

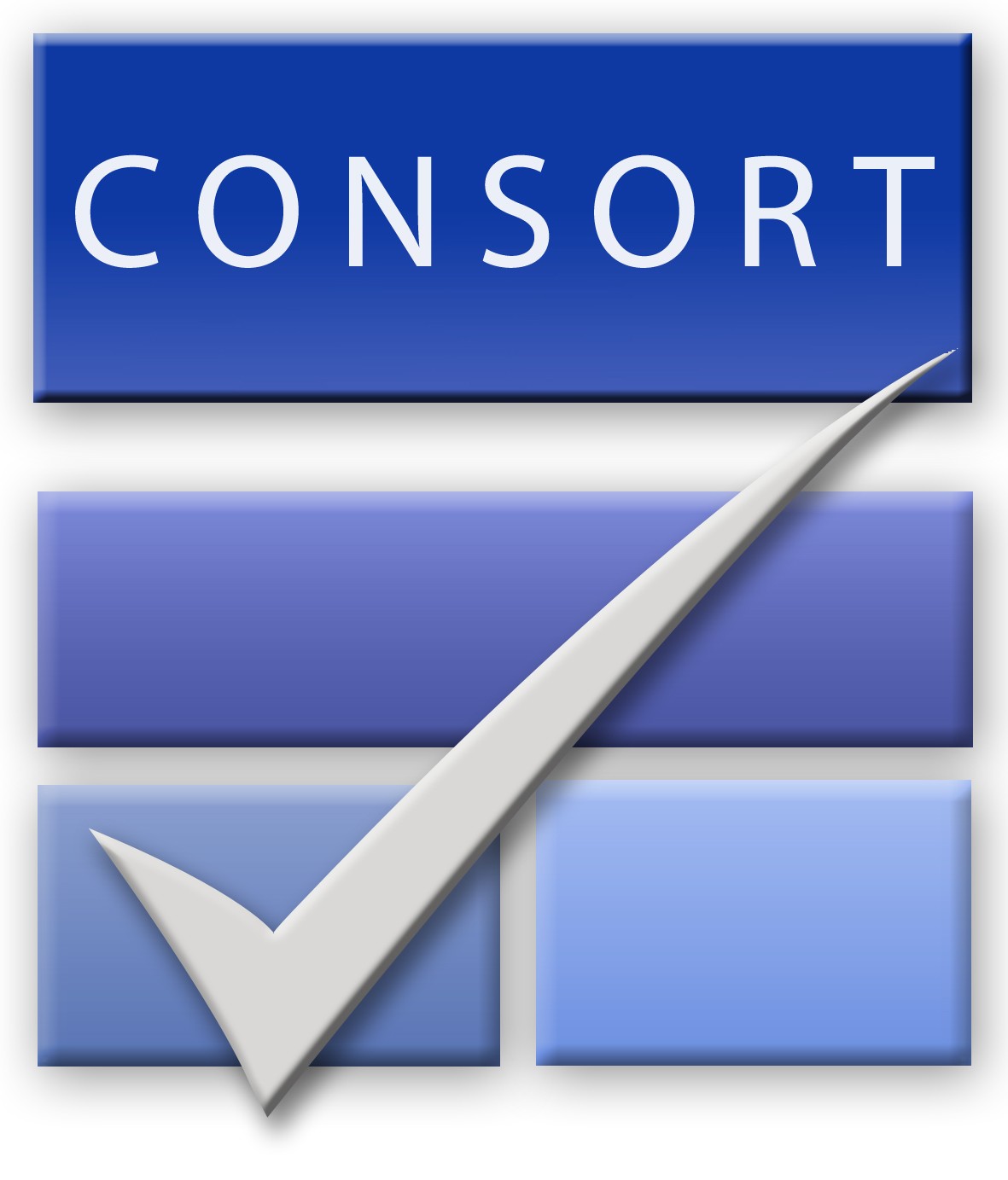
CONSORT 2010 checklist of information to include when reporting a pilot or feasibility trial*

| Section/Topic | Item No | Checklist item | Reported on page No |
| --- | --- | --- | --- |
| Title and abstract | | | |
|  | 1a | Identification as a pilot or feasibility randomised trial in the title | Page 1: Lines 3-5 |
|  | 1b | Structured summary of pilot trial design, methods, results, and conclusions (for specific guidance see CONSORT abstract extension for pilot trials) | Page 3: Lines 3-26. |
| Introduction | | | |
| Background and objectives | 2a | Scientific background and explanation of rationale for future definitive trial, and reasons for randomised pilot trial | Page 4: Lines 4-30 |
|  | 2b | Specific objectives or research questions for pilot trial | Page 5: Lines 3-8. |
| Methods | | | |
| Trial design | 3a | Description of pilot trial design (such as parallel, factorial) including allocation ratio | Page 5: Lines 12-17. |
|  | 3b | Important changes to methods after pilot trial commencement (such as eligibility criteria), with reasons | N.A. |
| Participants | 4a | Eligibility criteria for participants | Page 7: Lines 7-19. |
|  | 4b | Settings and locations where the data were collected | Page 7: Lines 1-4 |
|  | 4c | How participants were identified and consented | Page 6: Lines 19-29 |
| Interventions | 5 | The interventions for each group with sufficient details to allow replication, including how and when they were actually administered | Page 5: 20-31, Page 6: 1-5, Page 20: Table 1 |
| Outcomes | 6a | Completely defined prespecified assessments or measurements to address each pilot trial objective specified in 2b, including how and when they were assessed | Page 8 - 11 |
|  | 6b | Any changes to pilot trial assessments or measurements after the pilot trial commenced, with reasons | N.A. |
|  | 6c | If applicable, prespecified criteria used to judge whether, or how, to proceed with future definitive trial | N.A. |
| Sample size | 7a | Rationale for numbers in the pilot trial | N.A. |
|  | 7b | When applicable, explanation of any interim analyses and stopping guidelines | N.A. |
| Randomisation: |  |  |  |
| Sequence  generation | 8a | Method used to generate the random allocation sequence | N.A. |
|  | 8b | Type of randomisation(s); details of any restriction (such as blocking and block size) | N.A. |
| Allocation  concealment  mechanism | 9 | Mechanism used to implement the random allocation sequence (such as sequentially numbered containers), describing any steps taken to conceal the sequence until interventions were assigned | N.A |
| Implementation | 10 | Who generated the random allocation sequence, who enrolled participants, and who assigned participants to interventions | N.A. |
| Blinding | 11a | If done, who was blinded after assignment to interventions (for example, participants, care providers, those assessing outcomes) and how | N.A. |
|  | 11b | If relevant, description of the similarity of interventions | N.A. |
| Statistical methods | 12 | Methods used to address each pilot trial objective whether qualitative or quantitative | Page 8: Lines 30-32  Page 9: Lines 1-8  Page 10: Lines 22-31. Page 11: Lines 1-2. |
| Results | | | |
| Participant flow (a diagram is strongly recommended) | 13a | For each group, the numbers of participants who were approached and/or assessed for eligibility, randomly assigned, received intended treatment, and were assessed for each objective | Page 11: Lines 5-14  Page 20: Figure 1. |
|  | 13b | For each group, losses and exclusions after randomisation, together with reasons | Page 11: Lines 6-14  Page 20: Figure 1. |
| Recruitment | 14a | Dates defining the periods of recruitment and follow-up | Page 7: Lines 1-2 |
|  | 14b | Why the pilot trial ended or was stopped | N.A. |
| Baseline data | 15 | A table showing baseline demographic and clinical characteristics for each group | Page 21 and 22: Table 2 and 3. |
| Numbers analysed | 16 | For each objective, number of participants (denominator) included in each analysis. If relevant, these numbers  should be by randomised group | Page 11: Line 6-7 and 11-12, Pages 21-27: Table 2-7. |
| Outcomes and estimation | 17 | For each objective, results including expressions of uncertainty (such as 95% confidence interval) for any  estimates. If relevant, these results should be by randomised group | Page 12: Lines 2-6. Page 23-27: Table 4-7. |
| Ancillary analyses | 18 | Results of any other analyses performed that could be used to inform the future definitive trial | N.A. |
| Harms | 19 | All important harms or unintended effects in each group (for specific guidance see CONSORT for harms) | Page 12: Lines 15-20 |
|  | 19a | If relevant, other important unintended consequences | N.A. |
| Discussion | | | |
| Limitations | 20 | Pilot trial limitations, addressing sources of potential bias and remaining uncertainty about feasibility | Page 16: Lines 24-30  Page 17: 4-6 |
| Generalisability | 21 | Generalisability (applicability) of pilot trial methods and findings to future definitive trial and other studies | Page 14: Lines 3-11.  Page 14: Lines 16-22.  Page 15: Lines 14-17. |
| Interpretation | 22 | Interpretation consistent with pilot trial objectives and findings, balancing potential benefits and harms, and  considering other relevant evidence | Page 16: Lines 26-30. |
|  | 22a | Implications for progression from pilot to future definitive trial, including any proposed amendments | Page 16: Lines 8-20. |
| Other information | | |  |
| Registration | 23 | Registration number for pilot trial and name of trial registry | Page 3: Line 26. |
| Protocol | 24 | Where the pilot trial protocol can be accessed, if available | N.A. |
| Funding | 25 | Sources of funding and other support (such as supply of drugs), role of funders | Page 18: Lines 22-26. |
|  | 26 | Ethical approval or approval by research review committee, confirmed with reference number | Page 5: line 14. |

Citation: Eldridge SM, Chan CL, Campbell MJ, Bond CM, Hopewell S, Thabane L, et al. CONSORT 2010 statement: extension to randomised pilot and feasibility trials. BMJ. 2016;355.

*We strongly recommend reading this statement in conjunction with the CONSORT 2010, extension to randomised pilot and feasibility trials, Explanation and Elaboration for important clarifications on all the items. If relevant, we also recommend reading CONSORT extensions for cluster randomised trials, non-inferiority and equivalence trials, non-pharmacological treatments, herbal interventions, and pragmatic trials. Additional extensions are forthcoming: for those and for up to date references relevant to this checklist, see [www.consort-statement.org](http://www.consort-statement.org/).
